# Supplementary material for: Biofilms Positively Contribute to Bacillus amyloliquefaciens 54-induced Drought Tolerance in Tomato Plants
Source: Int J Mol Sci. 2019 Dec 12;20(24):6271. doi: 10.3390/ijms20246271 (PMC6940783; doi:10.3390/ijms20246271)
Supplement: Supplementary file 1 [file ijms-20-06271-s001.pdf]

Table 1.| Oligonucleotide primers used for qRT-PCR in this study.

Name	Sequence (5′–3′)
Actin-F	TGGGATGATATGGAGAAGATATGG
Actin-R	GGCTTCAGT TAGGAGGACAGGA
lea-F	GTCCCAATCTCCTCCAA
lea-R	CAGGGTAAT CGCATCAG
tdi65-F	CGTCCTCTGTTGTCCC
tdi65-R	AGTGAAGGCAATGAAGC
ltpg2-F	TACTGGACC GTTGAGCA
ltpg2-R	GGTGTTGTGGTGGTGTTA
nced1-F	AGGCAACAGTGAAACTTCCATCAAG
nced1-R	TCCATTAAAGAGGATATTACCGGGGAC

Table S2 | Drought tolerance efficacy of wild isolates under greenhouse conditions.

Strains	Possible Species	Drought Tolerance Efficacy*
−	−	+
Blt-11	*Acinetobacter* sp.	+ +
HS1-1	*Acinetobacter sp.*	+ + +
DS19	*Arthrobacter globiformis*	+
HS23-4	*Arthrobacter globiformis*	+ +
54	*Bacillus amyloliquefaciens*	+ + + +
2YN11	*Bacillus amyloliquefaciens*	+ +
5YN11	*Bacillus amyloliquefaciens*	+ +
HSSN09	*Bacillus amyloliquefaciens*	+ +
W13-3	*Bacillus cereus*	+ +
Blgb-12	*Bacillus cereus*	+ +
DS38	*Bacillus cereus*	+ +
HYN1	*Bacillus cereus*	+
HS10	*Bacillus lincheniformis*	+
31	*Bacillus megaterium*	+ +
3BS3	*Bacillus megaterium*	+ +
HS15	*Bacillus megaterium*	+ +
DS58	*Bacillus pocheonensis*	+
Ljb-10	*Bacillus pumilus*	+ +
DS22	*Bacillus pumilus*	+
YF31	*Bacillus sp.*	+ +
Lgn-13	*Bacillus sp.*	+ + +
Lgn-3	*Bacillus sp.*	+ +
Lgn-4	*Bacillus sp.*	+
DSSN12	*Bacillus subtilis*	+
Blgb-17	*Bacillus subtilis*	+ +
1BQN14	*Enterobacter asburiae*	+ +
YT8	*Enterobacter ludwigii*	+ +
3BJN7	*Enterobacter* sp.	+ +

Lgn-6	*Enterobacter* sp.	+ +

*Drought tolerance efficacy: Classification of isolates according to survival rate of tomato plant after 15 days of drought treatment and then 5 days of rewatering, where (+) 0-20%; (++) 21%-40%; (+++) 41%-60%; (+++)≥61%.

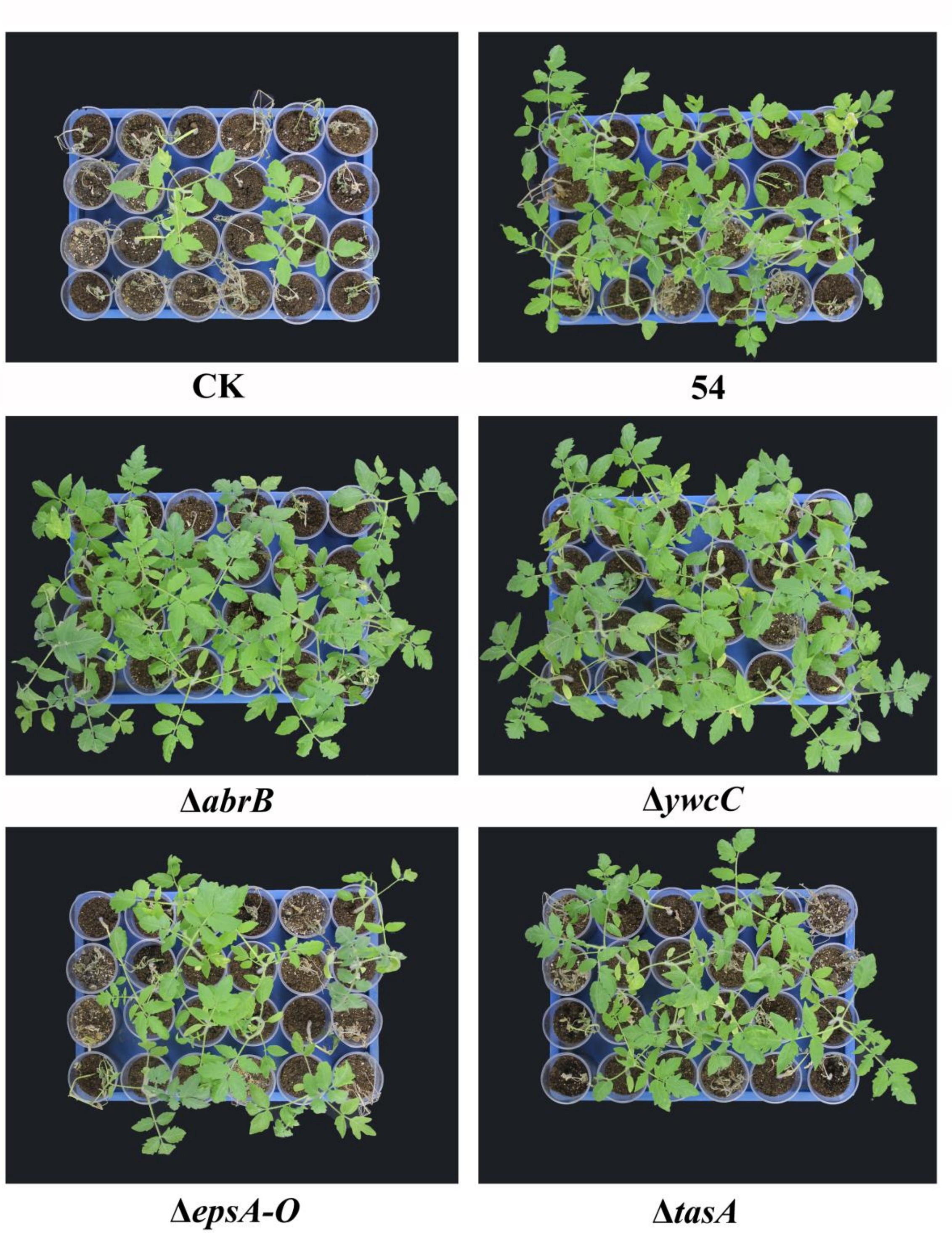

Figure S1. Biofilm formation is positivity involved in *B. amyloliquefaciens* 54-mediated drought tolerance of tomato plants. Photographs of each treatment were taken after 15 days of drought treatment and then 5 days of rewatering.
